# The Impact of Health and Wealth on Settlement Intention of Migrants: The Moderating Effect of Social Welfare

**DOI:** 10.3389/fpubh.2021.741812

**Published:** 2021-12-23

**Authors:** Xiao Zheng, Yaqing Xue, Yu Yin, Fang Dong, Jinghui Chang, Chichen Zhang

**Affiliations:** ^1^School of Public Health, Southern Medical University, Guangzhou, China; ^2^School of Health Management, Southern Medical University, Guangzhou, China; ^3^Department of Health Management, Nafang Hospital, Guangzhou, China; ^4^Institute of Health Management, Southern Medical University, Guangzhou, China

**Keywords:** migrants, settlement intention, health, wealth, social welfare

## Abstract

**Background:** With the rapid urbanization, citizenization of migrants is becoming the development tendency in China. It is significant to analyze the determining factors of the settlement intention of migrants.

**Methods:** The data we used were taken from the China Migrants Dynamic Survey (CMDS) in 2017. Multilevel mixed-effects logistic regression was used to analyze the relationship between air pollution, economic advantages, and settlement intention between different migrants and the moderating effect of social welfare.

**Results:** At the individual level, being female, married, urban and other ethnic, having higher education, older, and health associated with likelihood of settlement intention of migrants. Higher health education, social integration, and, have a health record were positively associated with the likelihood of settlement intention. Higher educated, urban areas, and Han migrants were willing to reduce their pursuit of health for economic development.

**Conclusion:** Health education and more social organizational participation can reduce the negative effect of air pollution and increase the positive effect of economic advantages on settlement intention of migrants. But, in less economically advantaged areas, it has no obvious effect. In the choice of health and wealth, the settlement intention of migrants shows difference, and unfairness and social welfare, in particular health education, can narrow this difference.

## Introduction

Intra-labor migration has provided positive impetus during China's economic reform and industrialization. With the development of the economy and the stabilization of the urbanization structure, the adjustment of China's migrant population policy has gone through three stages: gradually liberalizing peasants into cities, demanding fair treatment of the migrant population, and fully promoting citizenship (1). In the twenty-first century, the transformation of the social structure has advanced the process of urban-rural integration in China, deepening the unevenness between urban and rural areas in terms of economic and social welfare. For a long time, the size of the migrant population will continue to expand, while the characteristics of their settlement will also diversify. Benefiting from their subjective role, as the social environment changes, the Chinese have high bargaining power and can actively move to their favorite areas.

Many scholars have paid attention to the determinants of the migrants' settlement intention (2). There have been many observations of the mechanisms driving migration in research, with the “push-pull” hypothesis being one of the best-known explanations (1). The theory shows that the decision-making between settlement intention is based on the comparison of various factors among regions of migrants. The push-pull migration model is also applicable to this study, because the pull is the positive factor to improve the settlement intention mainly from the destination city, and the push is the negative factor to reduce the settlement intention mainly from the origin city. These factors include different levels of economic factors, environmental factors, and the public service level (1–3). In the research of migrants in China, the development of the push-pull theory has gone through two stages: firstly, in the 1980s, the choice to settle was more of a push-pull of structural factors, with government, market, and social influences being the underlying motivation for settlement (1). Secondly, with the advent of a floating society, the push-pull effect of individual level factors on the migrants has been reversed. These factors include objective factors, such as policy, economic, environmental, and social welfare factors. In addition to the objective factors mentioned above, personal ability factors, including the education level, vocational and technical skills, social networks, and the economic level of the family, also affect the settlement intention of migrants. The migration policy of cities is difficult to be changed, and we should give more attention to social welfare, environmental factors, and economic factors, which can help to improve the settlement intention of migrants.

### Economic Factors and Migration

Since Chinese economic reform, China's population movement has formed a distinctive spatial location from the countryside to the cities and towns, and from the central and western regions to the eastern coastal regions. Some scholars point out that China's mobile population is an economically oriented flow (4, 5). It is mainly from economically backward regions to economically developed regions, gradually forming the economically developed Pearl River Delta, Yangtze River Delta and Beijing-Tianjin-Hebei regions as the main inflow regions and the central and western, especially the less developed central regions as the main outflow regions (6). Some studies have shown that the level of urban economic development has a positive guiding effect on the settlement intention of migrants (7–10). A higher level of urban economic development means more development and employment opportunities for the migrants (11). The urban-rural dual structure theory and the new population migration theory emphasize the influence of economic income and expenditure on population mobility. The study of Ritchey emphasized the importance of economic income factor in the settlement of migrants (12). Some Chinese scholars found that, in intra-China mobility, the migrants' settlement intention is influenced by their socioeconomic status, including their income status and the occupation they are engaged in (13, 14). It has been suggested that the “pull” effect of higher economic levels and resource-rich regions on the residence of migrant populations is more pronounced (15). Studies by Han and Li (16, 17) found that the level of individual income expands with the expansion of city size and its economic level. Therefore, when considering the influence of economic dimensions on the residence of migrant population, income should not simply replace the overall economic level of the city, and the economic differences between the inflow and outflow areas are still important factors. Cities with higher levels of economic development are likely to be more attractive to flowing populations to settle down.

### Environmental Factors and Migration

Another view is that people will choose to take advantage of their opportunities to lead a better life and avoid undesirable risks and harms with economic development. Therefore, in addition to the inflowing places' economic factors and the environment, individual health and social welfare of the inflowing places also affect the migrants' settlement intention. Adger described the impact of economic development on migration, but he also suggested that the environment can have a meaningful impact on population migration (18). Warner found that climate change can bring about environmental pollution and cause the decline of environmentally dependent economic development societies, which can lead to population loss (19). Liu's study on internal migration in China showed that, when people inevitably suffer air pollution, they choose to move to cities with high air quality, thus securing a better living environment for themselves (3). A previous study found a negative correlation between air quality and labor supply (20). Therefore, the environment may be a vital factor in the settlement intention of the migrant population. This migration with the aim of a better environment is, ultimately, a health-seeking migration pattern (21).

### Social Welfare Factors and Migration

Tiebout's (22) theory of “voting with one's feet” suggests that capital, talent, and technology flow to administrative areas that provide more superior public services (22). Therefore, in the settlement choice of migrant population, social welfare including public services will have a positive impact on their intention to stay (23). Scholars such as Zhan (24) and Wang (25) conducted studies on health services, medical care, suitability and social integration aspects, and the intention to stay of the migrant population, and all found positive effects between the two. Fewer scholars have focused on the effect of health education in existing studies on social welfare on the migrant population's intention to settle down. Health education is increasingly accepted by the general public as an effective means to improve residents' health. Therefore, the study suggested that health education may influence the migrant population's intention to stay. This study regards health education as an indicator to evaluate social welfare.

The economic may be a pull factor and air pollution is a push factor. Based on this, we hypothesized that there are two existing migration patterns: wealth-driven migration and health-driven migration. Although China has proposed a series of policies to promote green development, the contradiction between economic development and environmental pollution still exists. The results of the correlation analysis between economic growth and environmental pollution in 31 provincial areas in China from 2008 to 2018 by Ye showed that there is a significant positive spatial correlation between economic growth and environmental pollution in China provincial areas (26). That is, the environmental pollution status is relatively higher in areas with faster economic development. Existing studies have addressed both health-based and wealth-based factors in the study of intent to settle among migrant populations within China, but fewer studies have focused on the conflicting nature and their specific manifestations in different populations. Liu's study confirmed the negative effect of environmental pollution on the willingness of the migrant population to settle down, and found that this negative effect was more obvious among highly educated, older, province-wide migrants, and rural residents (3), but the study did not focus on the interaction between air pollution and economic aspects and its endogeneity. Bo Li studied the effect of air pollution and income on the migrant population's willingness to stay and found that there was some conflict between the two. Bo Li investigated the impact of air pollution and income on the intention to stay of the mobile population and found that there was some conflict between the two. Specifically, non-rural populations had a higher pursuit of air quality, while rural and more spatially distant migrant populations had a higher pursuit of income (2). Although this study analyzed the interaction between income and health, the study did not consider the role of urban economic advantage and did not include the influence of social welfare factors.

In this study, we incorporate air pollution (push), urban economic advantage (pull), and social welfare (adjuster) into the “push-pull” theoretical model, explores the conflict between the two on the settlement intention of the migrants, and tests the moderating effect of social welfare factors. Second, individual differences reflect different bargaining powers for the same air pollution and urban economic advantage. Due to differences in individual characteristics, adaptability to the air pollution and urban economic advantage also shows varying systematic differences. So, we assessed specific impact of air pollution and urban economic advantage on the migrants.

Based on the above, the following hypotheses are proposed:

Hypothesis 1: The inflow place's economic advantage (the difference between the economic level of the inflowing places and the outflowing places) is the pull factor, which positively influences the residence choice of migrants and air pollution is the push factor, which negatively influences the settlement intention of migrants.

Hypothesis 2: Due to differences in individual characteristics, adaptability to air pollution and economic advantage also show varying systematic differences.

Hypothesis 3: Social welfare has a positive role in economic advantage and air pollution on the settlement intention of migrants.

## Methodology

### Sample

The data we used were taken from the China Migrants Dynamic Survey (CMDS) in 2017, conducted in all the provinces by the National Health and Family Planning Commission of People's Republic of China. CMDS focused on internal migrants, who were above 15 years old and have been living in destination cities for at least 1 month but without their household registration (Hukou) (excluding students, soldiers, and households where one of the spouses has the local hukou) (27). A three multiple-stage and Probability-Proportional-to-Size sample methods were used to size the samples. A total of 169,989 valid responses were obtained, which cover China's 31 provinces, autonomous regions, and municipalities. Detailed information about the purpose, design, sample, and the questionnaires of the CMDS is available in other studies (28, 29).

### Measurement

#### Dependent Variables

During the survey, migrants were asked “I would like to integrate with the local people and become one of them,” with answers being “totally agree,” “not sure yet,” “disagree,” and “totally disagree.” We recoded responses into two categories (1 = totally agree, 0 = others). The settlement intention of the population was 41.2%.

#### Independent Variables

(1) According to the purpose of the study, health status of individuals, the economic, air pollution status, public health services, and social inclusion in the inflow area were used as independent variables.

①We obtained data on city population density, gross regional product (GRP) per capita in “China Urban Statistics Yearbook 2018” (30). Some cities have missing GRP values, so we used the GRP of the province instead of the missing GRP of the city. There are only 8 cities with missing GRP values. Economic advantage of inflow areas = GRP of inflow area - GRP of the outflow area, divided into two categories: ≤ 0 is low economic advantage, considered as not pursuing economic development, and >0 is high economic advantage, considered as pursuing economic development.

②The commonly used measure of air quality in China is the Air Quality Index (AQI), which is determined by the concentrations of six criteria pollutants, including SO_2_, NO_2_, PM_2.5_ PM_10_, CO, and O_3_ (31). According to the Ambient Air Quality Index (AQI) Technical Provisions (Trial) (HJ 633–2012) (31), the air quality is classified into three classes according to the ranges of AQI values (<50: excellent, 51–100: good, >100: pollution). For this paper, we relied on the AQI values for April 2017, 1 month before the survey period of the CMDS Survey, to ensure that all migrants in the survey had experienced at least a month's worth of the observed air-quality conditions by the time they were surveyed. AQI values in this study were taken from the Chinese Air Quality Monitoring and Analysis website (https://www.aqistudy.cn/historydata/).

③A self-rated health was measured on a 5-point Likert scale by asking “how would you rate your current state of overall physical health?” Responses ranged from 1 to 5, representing “very poor,” “poor,” “fair,” “good,” and “excellent.” We constructed health as a binary variable. It was recoded into two categories: fair health or below (response = 1–3) and good health (response = 4–5). This transformation was the same as previous studies (32, 33).

④Public health services: Establishment of health records in the inflow area (1 = yes, 0 = no), missing value = 0. Health education index, which is determined by the number of education contents and methods people received. There are nine health education contents (27) and six health education methods (34); health education index ranges 0-15.

⑤Social organizational participation refers to the behavioral integration of the migrants in the inflow city, which is determined by 11 social and government activities that migrants participate in (27). Participation in this activity is counted as 1, not counted as 0, and the score ranges 0–11.

(2) We indicated gender, age, marital, education, hukou, and mobility range, and inflow cities of migrants were included in the model as control variables (Table 1).

**Table 1:** Descriptive statistics for original sample and variables unscaled.

**Variables**		**Scale**	**Mean (*SD*)**	***N* (%)**
Sex	Male	0		87,871 (51.7%)
	Female	1		82,118 (48.3%)
Marital	Other	0		31,906 (18.8%)
	Married	1		138,083 (81.2%)
Hukou	Other	0		37,434 (22.0%)
	rural	1		132,555 (78.0%)
Ethnic	other	0		15,997 (9.4%)
	Han	1		153,992 (90.6%)
Educational	Primary education	1		28,972 (17.0%)
	Secondary education	2		111,438 (65.6%)
	Higher education	3		29,579 (17.4%)
Age (years)			35.99 (11.08)	
	15–17	1		1,445 (0.9%)
	18–44	2		131,296 (77.2%)
	45–59	3		31,262 (18.4%)
	60–99	4		5,986 (3.5%)
Migration scope	Cross-provinces	1		83,790 (49.3%)
	Within-provinces	2		56,017 (32.9%)
	Within-city	3		30,182 (17.8%)
Health	No	0		30,299 (17.8%)
	Yes	1		139,690 (82.2%)
Health record	No	0		123,600 (72.7%)
	Yes	1		46,389 (27.3%)
Health education index			5.54 (5.22)	
Social organizational participation index			1.33 (1.59)	
GRP (yuan)			93,313.87 (36,501.55)	
Economic advantage			29,683.12 (41,658.37)	
	Low	0	≤ 0	59,883 (35.2%)
	High	1	>0	110,106 (64.8%)
AQI			79.94 (16.15)	
	Excellent	1		5,360 (3.2%)
	Good	2		148,791 (87.5%)
	Pollution	3		15,838 (9.3%)
Settlement intention	No	0		99,971 (58.8%)
	Yes	1		70,018 (41.2%)

### Analytical Strategies

Descriptive statistics were used to summarize characteristic variables related to individual and provincial levels (Table 1). We performed chi-square test and independent sample *t*-test on settlement intention among different groups. When a sample involves clustering by bigger geographical loci, the advantages of multilevel (or mixed effect) modeling over linear regression are apparent and fully elaborated elsewhere (35). We used multilevel logistic regressions to test Hypothesis 1, with individuals as the first-level units and cities as the second-level units:


$${\text{y}}_{\text{ij}}=\alpha_{\text{ij}}+\beta {\text{X}}_{\text{ij}}+\gamma {\text{Z}}_{\text{j}}+\text{λ}{\text{P}}_{\text{j}}+{\text{u}}_{\text{j}}+{\text{e}}_{\text{ij}}$$

where *y*_*ij*_ is a binary value of settlement intention for the *i*th individual in *j*th city, *P*_*j*_ is the characteristic in *j*th city, *X*_*ij*_ is a vector of individual-level variance, and *e*_*ij*_is the error term for individuals. Firstly, we ran an empty model of multilevel logistic regressions (Model 2), serving as a baseline comparison. Second, we contained only city-level variables, including GRP, economic advantage, air quality, and population density in the Model 3. Third, we added health and social welfare indicators, including health status, the establishment of a health record in the inflowing place, health education, and social organizational participation index in Model 4. Finally, based on Model 3 and Model 4, we added control variables, including sex, marital, hukou, ethnic, educational, and migration scope to Model 5.

For Hypothesis 2 that expects the impact of air quality and urban economic advantage to be moderated by educational, ethnic, and hukou, we included cross-level interaction terms and tested their significance. We tested two-way interactions (air quality × urban economic advantage) for the three migration groups capturing this way, the intersections across the categories of interest (air quality × urban economic advantage × educational, air quality × urban economic advantage × ethnic, air quality × urban economic advantage × hukou).

For Hypothesis 3, we tested two-way interactions to analyze the moderating effects of health education and social organizational participation on the relationship of air pollution and settlement intention (air pollution × health education, air pollution × social organizational participation) and the relationship of urban economic advantage and settlement intention (urban economic advantage × health education, urban economic advantage × social organizational participation) of migrants. All of the statistics in this paper are produced in STATA 17.0. The significant level was set at *p* < 0.05.

## Results

### Settlement Intention Among Different Groups

The chi-square test was used to analyze the categorical variables. The results show that there were differences in the settlement intention among different migrants (*p* < 0.01). The migrant whose migration scope is within a city has the highest settlement intention (46.3%). The migrants who are healthy (41.5%) and have a health record (49.3%) show higher willingness to settle. The better the air quality, the stronger the willingness of migrants to settle down (48.3% > 41.5% > 35.5%) (see Table 2).

**Table 2:** Comparison of settlement intention among different groups.

**Variables**		**Settlement intention** ***N*** **(%)**			***P***
		**No**	**Yes**	***X^**2**^***	
Sex	Male	52,319 (59.5)	35,552 (40.5)	40.06	<0.001
	Female	47,652 (58.0)	34,466 (42.0)		
Martial	Other	20,461 (64.1)	11,445 (35.9)	458.7	<0.001
	Married	79,510 (57.6)	58,573 (42.4)		
Hukou	Other	18,665 (49.9)	18,769 (50.1)	1,600.00	<0.001
	Rural	81,306 (61.3)	51,249 (38.7)		
Ethnic	Other	9,214 (57.6)	6,783 (42.4)	10.71	0.01
	Han	90,757 (58.9)	63,235 (41.1)		
Educational	Primary education	17,767 (61.3)	11,205 (38.7)	1,000.00	<0.001
	Middle education	67,261 (60.4)	44,177 (39.6)		
	Higher education	14,943 (50.5)	14,636 (49.5)		
Migration scope	Cross-provinces	52,286 (62.4)	31,504 (37.6)	930.27	<0.001
	Within-provinces	31,479 (56.2)	24,538 (43.8)		
	Within-city	16,206 (53.7)	13,976 (46.3)		
Health	No	18,306 (60.4)	11,993 (39.6)	39.33	<0.001
	Yes	81,665 (58.5)	58,025 (41.5)		
Health record	No	76,432 (61.8)	47,168 (38.2)	1,700.00	<0.001
	Yes	23,539 (50.7)	22,850 (49.3)		
Economic advantage	Low	33,153 (55.4)	26,730 (44.6)	453.56	<0.001
	High	66,818 (60.7)	43,288 (39.3)		
AQI	Excellent	2,773 (51.7)	2,587 (48.3)	305.77	<0.001
	Good	86,989 (58.5)	61,802 (41.5)		
	Pollution	10,209 (64.5)	5,629 (35.5)		

An independent sample *t*-test was used to analyze continuous variables, and the results showed that older migrants exhibit higher settlement intention (36.82 years old > 35.41 years old). Health education (6.33 > 4.99) and social organizational participation (1.55 > 1.17) were positively correlated with migrants' settlement intention. The level of GRP and economic advantages was positively correlated with settlement, implying that people like to move to rich places (*p* < 0.001). Residents may gravitate toward places with better air quality (AQI, 79.13 <80.5) (see Table 3).

**Table 3:** The relationship between characteristics of migrants and settlement intention.

**Variables**	**Settlement intention**		**Mean difference**	***t***	***P***
	**No**	**Yes**			
Age (year)	35.41	36.82	−1.41	−25.93	<0.001
Health education	4.99	6.33	−1.34	−52.51	<0.001
Social organizational participation	1.17	1.55	−0.37	−47.91	<0.001
GRP (yuan)	93,720.99	91,521.95	2,199.04	12.91	<0.001
Economic advantages	31,099.66	27,660.59	3,439.06	16.77	<0.001
AQI	80.5	79.13	1.37	17.20	<0.001

### Influencing Factors of Migrants' Settlement Intention

To gain greater understanding of the effects of health and wealth on settlement intention in migrants, we incorporated health factors, wealth factors, and social welfare factors into the regression equation and established a binary logistic regression model and a mixed effect logistic regression model. Due to the differences in the units and magnitudes of the independent variables, we cannot identify the relative influence of each variable on the probability of settlement intention (36). Normalization of units was carried out to identify the relative effect of continuous variables, including scores of health education, social organizational participation, GRP, economic advantages, and AQI, on the probability of settlement intention.

Model 1 is a binary logistic regression model, in which the spatial heterogeneity and agglomeration effects of cities were not considered. It was found that, adjusted for demographic characteristics, the healthier, more health education, and the higher social organizational participation of the migrants have a higher settlement intention. Air quality and the higher economic advantage of cities were positively associated with the settlement intention of the migrants. Model 2 is the intercept-only model, serving as a baseline comparison. Model 3 contained only city-level variables, including GRP, economic advantage, air quality, and population density in the inflow cities. Accounting for urban heterogeneity, only the economic advantage (0.96, *p* < 0.001) of the inflow cities associated with the settlement intention of the migrants. Model 4 incorporated health and social welfare indicators, including health status (1.24, *p* < 0.001), the establishment of a health record (1.18, *p* < 0.001) in the inflow cities, health education (1.16, *p* < 0.001), and social organizational participation (1.20, *p* < 0.001). The results showed that all the above factors positively associated with the willingness to settle down of the migrants (Table 4).

**Table 4:** Multilevel mixed-effects logistic regression of settlement intention of migrants.

	**Model 1**	**Model 2**	**Model 3**	**Model 4**	**Model 5**
**Sex (male)**					
Female	1.12 (1.10–1.14)				1.09 (1.06–1.11)
**Marital (other)**					
Married	1.19 (1.15–1.22)				1.14 (1.11–1.17)
**Hukou (other)**					
Rural	0.75 (0.73–0.77)				0.83 (0.81–0.85)
**Ethnic (other)**					
Han	0.92 (0.89–0.95)				0.92 (0.88–0.95)
**Educational (primary)**					
Middle	1.08 (1.05–1.12)				1.05 (1.02–1.08)
Higher	1.45 (1.39–1.51)				1.29 (1.24–1.35)
Age	1.02 (1.01–1.02)				1.01 (1.01–1.02)
**Migration scope**					
Within-province	1.21 (1.18–1.24)				1.23 (1.20–1.27)
Within-city	1.30 (1.26–1.35)				1.41 (1.36–1.48)
**Health (no)**					
Yes	1.19 (1.16–1.23)			1.11 (1.08–1.15)	1.24 (1.21–1.28)
**Health record (no)**					
Yes	1.22 (1.19–1.25)			1.21 (1.18–1.24)	1.18 (1.15–1.21)
**Health education**	1.16 (1.14–1.17)			1.17 (1.16–1.18)	1.16 (1.15–1.18)
**Social integration**	1.17 (1.15–1.18)			1.20 (1.19–1.21)	1.19 (1.18–1.20)
**GRP**	0.93 (0.91–0.94)		1.03 (0.94–1.13)	1.03 (0.94–1.13)	1.01 (0.92–1.10)
**Economic advantages**	1.04 (1.02–1.06)		0.96 (0.94–0.98)	0.98 (0.96–0.99)	0.98 (0.96–1.00)
**Economic advantages**
High	0.95 (0.92–0.99)		0.88 (0.85–0.91)	0.90 (0.87–0.93)	1.04 (0.99–1.08)
**AQI**	0.99 (0.98–1.00)		0.94 (0.85–1.03)	0.92 (0.84–1.01)	0.92 (0.84–0.99)
**AQI (excellent)**					
Good	0.85 (0.80–0.91)		0.88 (0.50–1.56)	0.91 (0.52–1.59)	0.89 (0.52–1.52)
**Pollution**	0.67 (0.61–0.72)		1.02 (0.49–2.12)	1.07 (0.52–2.21)	1.06 (0.54–2.12)
**Population density**	1.03 (1.02–1.04)		0.99 (0.86–1.13)	0.97 (0.85–1.11)	0.99 (0.87–1.13)
Random effect					
Intercept		0.81	0.81	0.79	0.75
AIC, BIC		218,373, 218,393	218,201, 218,292	214,790, 214,921	213,041, 213,262

*The results of OR (95% CI) are reported in the table*.

Model 5 adjusted for individual-level variables. At the individual level, we found that, being female (1.09, *p* < 0.001), married (1.14, *p* < 0.001), urban and other ethnic, and having higher education (1.29, *p* < 0.001) were associated with likelihood of settlement intention. Older and healthy migrants more likely to settle down. For social welfare indicators, higher health education (1.16, *p* < 0.001), more social organizational participation (1.19, *p* < 0.001) and have a health record (1.18, *p* < 0.001) were positively associated with likelihood of settlement intention. After control for individuals' and cities' differences, we found economic advantages were not associated with settlement intention among migrants. A standard unit increase in air particulate matter was associated with 0.92 times lower likelihood of settlement intention. It means that air pollution is negatively associated with settlement intention among migrants (see Table 4).

### The Interaction Effects of Economic Advantage and Air Quality on the Settlement Intention of Migrants

**Figure 1:**
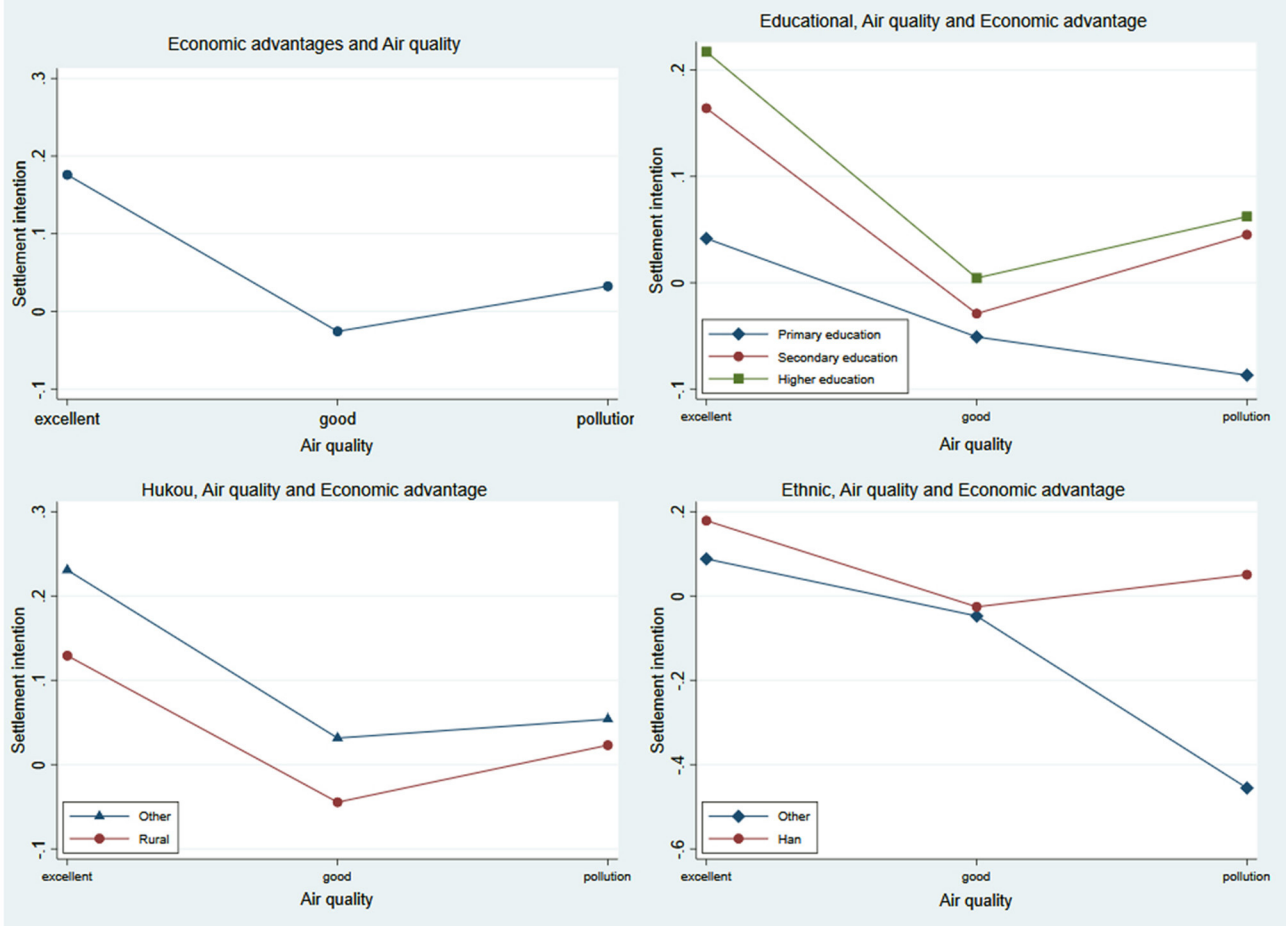
The interactive effects of air quality and economic advantages on settlement intention of migrants.

To assess the robustness of the above results, we first analyzed the interaction effect of economic advantage and air quality on the settlement intention of the migrants (air quality × economic advantage). The results show that the marginal effect of air quality on the settlement intention of the migrants was influenced by the economic advantage. When the air quality was excellent, the standardized economic advantage value increased by 1 unit, the settlement intention of migrants increased by 0.17 (*p* < 0.001), while the settlement intention decreased by 0.03 (*p* < 0.001) when the air quality was good. When the environment was polluted, the settlement intention increased by 0.03, but it is not statistically significant (*p* > 0.05). The results indicated that the effect of health on the settlement intention of migrants was more significant than wealth (Figure 1).

**Figure 2:**
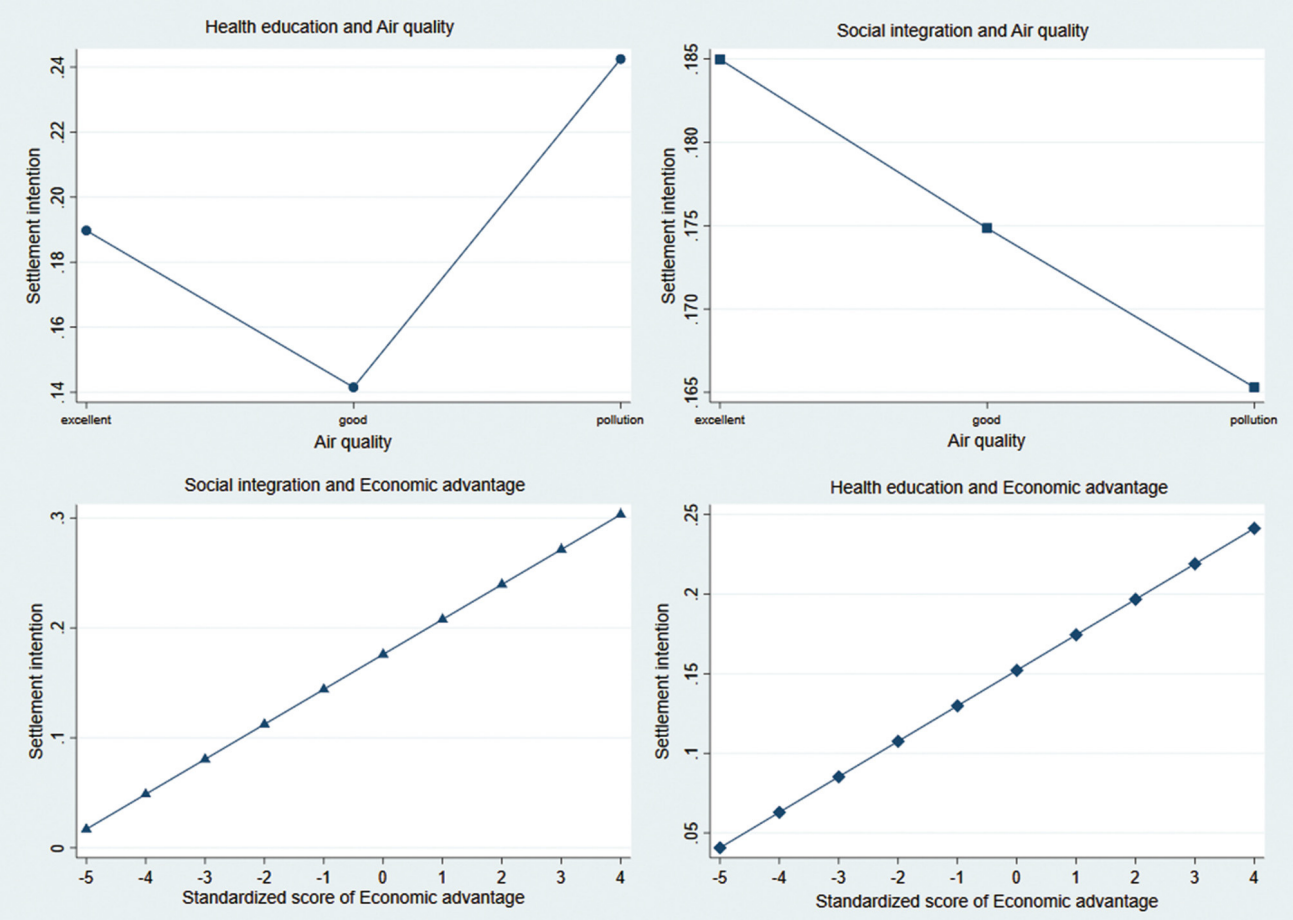
The interactive effects of health education, social organizational participation, air quality, and economic advantages on settlement intention of migrants.

We further investigated whether health- or wealth-related interest in settling down varied across different groups. Figure 2 shows the marginal effects when the group variables are fixed at different values. (1) **air quality** **×** **urban economic advantage** **×** **educational**: Education was positively associated with the settlement intention of migrants. When the air quality was excellent, economic advantage was positively associated with the settlement intention of migrants with higher education, and the marginal effects of settlement intention were 0.21 for higher education, 0.16 for secondary education, and 0.04 for primary education. The economic advantage was associated with settlement intention of migrants with secondary education (0.05) and higher education (0.06), when air quality was polluted, it was not statistically significant (*p* > 0.05). The results suggested that migrants with higher education were willing to sacrifice their health for economic development compared to those with lower education. (2) **Air quality** **×** **urban economic advantage** **×** **hukou:** The settlement intention was lower for the rural migrants than others. Excellent air quality was positivity associated with settlement intention at a significant greater magnitude among the urban migrants (0.23) than the rural migrants (0.13). But when the air quality was good, settlement intention in the urban migrants increased by 0.03 due to economic advantage but decreased in the rural migrants (0.04). The results suggest that wealth has a positive effect on the settlement intention of the urban migrants, while the rural migrants were affected by health. (3) **Air quality** **×** **urban economic advantage** **×** **ethnic**: According to Model 5, ethnic majority migrants have a higher settlement intention. However, the interaction results showed that economic advantage was positively associated with the settlement intention of the Han migrants, when air quality is excellent. While, when the air was polluted, economic advantage was negatively associated with the settlement intention of the ethnic minority migrants (0.45), and there was a non-significant increase in the settlement intention of the Han migrants (0.05, *p* > 0.05). In summary, the settlement intention of ethnic minority was influenced by health, while the Han migrants were influenced by wealth (Figure 1).

### The Interactive Effects of Health Education, Social Organizational Participation, Air Quality, and Economic Advantages on Settlement Intention of Migrants

To analyze the effect of social welfare on the settlement intention of the migrants at different levels of air quality and economic advantages, we constructed the interaction models of health education and social organizational participation with air quality and economic advantage, respectively.

(1) **Air quality** **×** **health education:** Health education increased the settlement intention of migrants in areas with different kinds of air quality. When air quality was polluted, health education increased the settlement intention of migrants by 0.24; when air quality was excellent and good, settlement intention was increased by 0.19 and 0.14. Therefore, health education can reduce the negative effect of air pollution on the settlement intention of the migrants. (2) **Air quality** **×** **social organizational participation:** When the standard value of economic advantage was < -4, health education was not correlated with the settlement intention among the migrants (*p* > 0.05). Furthermore, with greater economic advantage, higher health education was associated with settlement intention of the migrants. Health education increased the positive effect of economic advantage on the settlement intention of the migrants, but, in areas with low-economic advantage, there was no significant effect. (3) **Economic advantage** **×** **health education:** Same as health education, social organizational participation increased the settlement intention of the migrants in areas with different types of air quality, but there was a least effect in air pollution areas (excellent air quality: 0.18, good air quality: 0.17, air pollution: 0.16). The results indicated that social organizational participation can reduce the negative effect of air pollution on the settlement intention of the migrants. (4) **Economic advantage** **×** **social organizational participation:** The greater the economic advantage, the stronger the effect of social organizational participation on the intention to settle down of the migrants, but there was no significance at the standard value of economic advantage < -4. That is, social organizational participation increased the settlement intention of the migrants, but no significant advantage was found in areas with low economic advantage (see Figure 2).

## Discussion

Existing studies have addressed both macro and micro dimensions of settlement intention of migrants, but less focus on the relationships between the influencing factors. Based on the push-pull theory, this study examines the factors of the migrants' settlement intention, analyzing the pulling effect of the economic advantages, the pushing effect of air quality, and the moderating effect of social welfare factors. We hope to provide more sufficient evidence for the research on the residence of the migrant population and help relevant policymakers to formulate policies to improve the settlement rate of the migrant population. First, this study verifies the differences in the settlement intention among migrants from different groups. The study finds that female, married, minority, high age, non-farm hukou, and migrants with a closer mobility range are more willing to settle in the inflow cities, which is the same as the results of previous studies (2, 27).

Second, we found that the migrants often faced a trade-off between economic advantages and air quality in their settlement intention. This result is same as previous studies (2). In varying degrees, the economic advantage of the inflow cities would offset the negative effect of air pollution on the migrants' settlement, and the effect differs among different populations. Higher-educated migrants tended to tolerate poorer air quality to obtain higher economic advantage, which is the same as the results of Liu's study (3). Zhang's study concluded that the effect of air pollution on well-being was more pronounced among people with low education, and Kan's study (37) found that the effect of air pollution on the migrants with low education was more obvious compared to migrants with high education. These results may be that those with low-educated people are less aware of air pollution precautions. It also explains the fact that the highly educated people have better protective measures against air pollution. This study confirms that the settlement of highly educated migrants is less affected by air pollution and more attracted by economic advantages. The settlement intention of rural migrants is more likely to be negatively affected by air pollution, while urban migrants are more likely to be influenced by economic advantage. This results are same as the findings of Liu and Fan (3). A study on the population movement's effect on air pollution found that population movement in China reduced PM2.5 exposure levels, especially in rural areas (38). Liu pointed out that urban air quality is lower than rural, and urban residents have adapted to the effects of air pollution, which leads them to be less influenced by this factor in their settlement choices (3). However, “megacities,” such as Beijing and Shanghai, have increased PM2.5 exposure due to population movement (38). The findings suggest that rural Chinese migrants who move to cities are still economically weak and sacrifice their well-being for higher income opportunities. They have not yet crossed the income threshold, so they prioritize settling in a place with a safer environment rather than a place with a higher income but an unsafe environment (2). There is a two-way relationship between population movement and air pollution, and environmental management as a goal of urban development is equally important for attracting migrants to settle and promoting population health. In general, ethnic migrants' settlement is higher than Han migrants. But ethnic migrants' settlement intention was decreased due to the air pollution and was not affected by economic advantage. In contrast, Han migrants tended to tolerate poorer air quality to obtain higher economic advantage. Compared to Han areas, ethnic minority areas are smaller and have a relatively better environment, so ethnic minorities are more sensitive to the air quality (39, 40). Besides, social organizational participation is an important factor in the settlement of ethnic migrants.

Finally, we found that the establishment of health records, more health education, and the involvement of social organizations can increase the settlement intention of the migrants. This result reflects the attraction of health to the migrants (41). We also confirm the moderating role of health education and social organization participation in the relationship between air quality, economic advantage, and the settlement intention of the migrants. On the one hand, health education and social organizational participation are effective in increasing the positive effect of economic advantage on the migrants' settlement intention. The migrants tend to settle in cities with greater economic advantages, more health education, and better social organizational participation. On the other hand, health education and social organizational participation can reduce the negative effects of air pollution. Health education has offsetting effect on air pollution's negative effects, because better health education can help the migrants establish a healthy lifestyle and obtain more health protection measures. The study of Zhao (42) confirmed that health education has a more significant enhancing effect on the migrant population's health. Therefore, health education can help the migrants to respond to the health hazards caused by air pollution and increase their settlement intention. Although social organizational participation can reduce the negative effect of air pollution, the effect becomes lower when air pollution is getting serious.

There are some limitations to the study. Due to data limitation, the dependent variable in our paper is the intention instead of the real decision to settle down. For example, when considering wealth factors and city-level economic factors, economic advantage is used and personal income is missing. Thus, the evidence we have provided may only capture an approximate influence on settlement intention because of air pollution and economic advantage, and health education. In addition, our study reveals a close relationship between air pollution, economic advantage, and settlement intention among different migrants. We also identified the important role of health education and social participation for migrants. This result suggests that the government can attract migrants through urban construction, in addition to direct migration policies. However, we cannot determine the relationship between air pollution, economic advantages, health education, and macro immigration policy in this study, which needs to be supplemented with more data in further research.

## Conclusion

Based on existing studies, this paper analyzes the conflicting effects of health and wealth on the settlement intention of migrants and the differences among different groups. In varying degrees, the migrants tended to tolerate poorer air quality to obtain higher economic advantage. Health education and social organizational participation are effective in increasing the positive effect of economic advantage and in decreasing the negative effect of air pollution. In order to attract migrants, green and environmentally friendly industries should be encouraged in the cities of emerging industrializing countries. At the same time, basic public health services should be improved and more health education should be provided for migrants, encourage them to participate in social activities, to further improve the livability of cities for both residents and migrants. It also shows that, in the problem of migrants' settlement, it is unfair to rural areas, people with low education, and minority populations. These groups are relatively vulnerable, and they do not receive enough income, so they prioritize settling in a place with a safer environment rather than a place with a higher income. In the migrant population's management, the government and related policymakers should provide more policy support for such populations to help them improve their economic levels and health status. Besides, the authorities are supposed to provide health education and other social welfare as an essential means to improve the migrants' health and ultimately achieve the migrant population's citizenship.

## Data Availability Statement

The datasets presented in this study can be found in online repositories. The names of the repository/repositories and accession number(s) can be found below: http://www.ldrk.org.cn/.

## Author Contributions

CZ was involved in conceptualizing and designing the study, and drafting of initial version of the manuscript. XZ was involved in data analysis and in drafting the manuscript. YX and YY carried data collection and analysis. FD and JC were involved in formal analysis. All the authors have read and agreed to the published version of the manuscript.

## Funding

This study was supported by National Natural Science Foundation of China (71874104) and Key Laboratory Development Project for Philosophy and Social Sciences in Guangdong (G620369695), National Key R&D Program of China under Grant (2020YFC2006400).

## Conflict of Interest

The authors declare that the research was conducted in the absence of any commercial or financial relationships that could be construed as a potential conflict of interest.

## Publisher's Note

All claims expressed in this article are solely those of the authors and do not necessarily represent those of their affiliated organizations, or those of the publisher, the editors and the reviewers. Any product that may be evaluated in this article, or claim that may be made by its manufacturer, is not guaranteed or endorsed by the publisher.
